# Editorial: New insights into brain imaging methods for rehabilitation of brain diseases

**DOI:** 10.3389/fnins.2024.1397293

**Published:** 2024-03-20

**Authors:** Bin Hu

**Affiliations:** Department of Clinical Neurosciences, Hotchkiss Brain Institute, Cumming School of Medicine, University of Calgary, Calgary, AB, Canada

**Keywords:** neurorehabilitation, music therapy, brain imaging interventions, TMS, EEG, fMRI

This editorial on the Research Topic of Neurorehabilitation aims to concisely present the contributions of each study within the broader context of neurorehabilitation research, showcasing the collaborative effort to advance the field.

First of all the landscape of neurorehabilitation is enriched by the pioneering studies of Wang et al., Zou et al., Yang et al., and their contemporaries, who collectively push the boundaries of our understanding and treatment capabilities for neurological conditions. These researchers harness a variety of innovative methods, from functional near-infrared spectroscopy (fNIRS) to machine learning algorithms, to explore cognitive impairment, motor function recovery, and beyond.

Wang et al.'s nomograms for predicting cognitive impairment post-TBI set a precedent for personalized patient care. Zou et al. and Yang et al. further this narrative by utilizing fNIRS to investigate cognitive impairment and the therapeutic potential of sensory tasks in stroke rehabilitation. The collaborative work of Chen, Zhang, et al. introduces a nuanced understanding of interhemispheric imbalance, advocating for individualized neuromodulation strategies.

In parallel, Lin et al. review the promising effects of noninvasive brain stimulation on dual-task performance, whereas Xiao et al. and Song et al. delve into the realms of music therapy and ultrasonic neuromodulation, revealing new therapeutic avenues. The studies by Yang et al. and Xia et al. emphasize the role of alternative therapies like acupuncture and the physiological insights from acoustic startle priming, broadening the scope of neurorehabilitation strategies.

Moreover, Liu L. et al.'s development of a rodent-specific TMS coil and Zhang et al.'s identification of biological markers for post-stroke depression exemplify the integration of technology and biology in research. Further contributions from Liu S. et al., Chen, Huang, et al., and Zhao et al. focus on the practical applications of these findings in clinical settings, from improving balance and gait in cerebral infarction patients to enhancing diagnostic accuracy for Alzheimer's disease.

The comprehensive analysis by Zhou et al. of oxidative stress in ischemic stroke underlines the importance of addressing biochemical pathways in recovery. Finally, Shen et al.'s study on the effects of focal muscle vibration therapy showcases the potential of physical interventions in activating brain regions for motor function improvement.

Collectively, these studies not only underscore the importance of multidisciplinary approaches in neurorehabilitation but also highlight the potential for significant advances in patient outcomes through the integration of innovative research and clinical practice.

Below I will further comment on the unique contributions made by different groups of contributing authors.

The study by Wang et al. investigates the prediction of cognitive impairment in patients in with mild-to-moderate traumatic brain injury (TBI) through clinical and radiological parameters. They developed nomograms based on identified risk factors, such as age, Glasgow Coma Scale score, education level, hyperlipidemia, temporal lobe contusion, traumatic subarachnoid hemorrhage, very early rehabilitation, and ICU admission, to predict cognitive impairment at 3 and 12 months post-injury. The nomograms demonstrated good discriminative ability, indicating their potential utility in clinical management and intervention planning for TBI patients.

The study by Zou et al. investigates the functional connectivity in post-stroke cognitive impairment patients using functional near-infrared spectroscopy (fNIRS). It compares resting-state functional connectivity among patients with post-stroke cognitive impairment, patients without cognitive impairment, and healthy controls. The findings reveal that patients with cognitive impairment exhibit significantly decreased interhemispheric and intra-right hemispheric functional connectivity, suggesting that fNIRS could be a valuable tool in identifying patients at risk of cognitive impairment following a stroke.

The study by Yang et al. focuses on the impact of a bilateral plantar contact task on dorsolateral prefrontal activation in cerebral infarction patients, under both open and closed eye conditions. Using functional near-infrared spectroscopy (fNIRS), the research found that performing the task with eyes open significantly influenced dorsolateral prefrontal cortex activation, especially on the paralyzed side. These findings suggest that cognitive-motor therapies, which activate cognitive control brain regions through sensory tasks, might be effective in rehabilitating motor functions in cerebral infarction patients.

The study by Chen, Huang, et al. explores the use of functional near-infrared spectroscopy (fNIRS) and transcranial magnetic stimulation (TMS) to assess interhemispheric imbalance and its correlation with motor function recovery after stroke. The research demonstrates that combining TMS and fNIRS metrics provides insights into the role of hemispheric activity in recovery, suggesting potential for developing individualized neuromodulation strategies for stroke rehabilitation.

The study by Lin et al. systematically reviews the effects of noninvasive brain stimulation (NIBS) on dual-task performance across different populations, including healthy young adults, older adults, and individuals with Parkinson's disease (PD) and stroke. The research assesses both transcranial direct current stimulation (tDCS) and repetitive transcranial magnetic stimulation (rTMS), focusing on their impact on balance, mobility, and cognitive function under single-task and dual-task conditions. The findings suggest promising effects of tDCS and rTMS in improving dual-task walking and balance performance across these diverse groups, although the heterogeneity of the studies and limited data prevent definitive conclusions.

In the groundbreaking study conducted by Xiao et al., the team delves into the realm of music therapy, showcasing its profound impact on patients in a minimally conscious state (MCS). This ingenious and high-quality original research not only sheds light on the significant improvements in autonomic nervous system indicators and Glasgow Coma Scale scores but also leads to a pivotal change in clinical practice. By comparing the outcomes among patients receiving music therapy to those provided with familial auditory stimulation or standard care, Xiao et al. reveal the potential of music therapy as a superior rehabilitative intervention. The research convincingly argues for the integration of music therapy into the standard neurorehabilitation protocol, marking a transformative step forward in enhancing the quality of life and recovery prospects for MCS patients. This study stands as a testament to the power of innovative therapeutic approaches in revolutionizing patient care in neurorehabilitation.

The study by Song et al. explores the potential of ultrasonic neuromodulation mediated by mechanosensitive ion channels, highlighting its non-invasive, high-resolution, and targeted approach as an alternative to drug-based and invasive therapies. This perspective outlines the roles of various mechanosensitive ion channels like Piezo and TRP channels in neuronal excitability and biological effects induced by ultrasound, emphasizing the need for deeper understanding and further research in this promising field.

The study by Yang et al. focuses on the effects of acupuncture on brain function in patients with Cerebral Small Vessel Disease Cognitive Impairment (CSVDCI). It utilized amplitude of low-frequency fluctuation (ALFF) analysis in a randomized control trial setting to assess changes in brain activity. The findings suggest that acupuncture treatment significantly modulates the functional activity of certain brain regions in CSVDCI patients, pointing toward its potential utility in enhancing cognitive functions through specific neural mechanisms.

The study by Xia et al. examines the effect of acoustic startle priming (ASP) on the activation of the reticulospinal tract (RST) and its influence on motor response time. Through an innovative approach using functional near-infrared spectroscopy (fNIRS), they observed increased activation in the right dorsolateral prefrontal cortex and changes in frontoparietal activity during ASP tasks. These findings suggest the involvement of the right dorsolateral prefrontal cortex and frontoparietal network in regulating the StartleReact effect and RST facilitation, providing new insights into the neural mechanisms underlying motor control and facilitation.

The study by Liu L. et al. introduces a novel rodent-specific transcranial magnetic stimulation (TMS) coil equipped with a custom shielding device to enhance focal stimulation. This development aims to improve the spatial focus of TMS in animal models, thereby facilitating more precise neuroscientific research. Their findings demonstrate that the shielding device significantly narrows the stimulated area without compromising the intensity of the core magnetic field, potentially enabling more targeted brain area stimulation in rodent studies of neurological disorders.

The study by Zhang et al. focuses on identifying biological features associated with post-stroke depression (PSD) through machine learning algorithms. By analyzing gene expression profiles and employing weighted gene co-expression network analysis (WGCNA), the research identifies key genes and metabolic pathways linked to PSD. The findings highlight the potential of specific genes, SDHD and FERMT3, as diagnostic and therapeutic biomarkers for PSD, offering new avenues for early diagnosis and treatment strategies in stroke patients.

The study by Liu S. et al. explores the correlation between balance function, plantar pressure distribution, and gait parameters in patients with cerebral infarction in the basal ganglia region. It focuses on analyzing how balance function influences plantar pressure and hemiplegic gait, utilizing the Berg Balance Scale among other measures. The findings indicate a significant relationship between balance function and various gait and pressure parameters, suggesting that interventions aimed at improving balance could enhance gait performance and safety in stroke rehabilitation.

The study by Chen, Huang et al. analyzed spontaneous brain activity in patients with cerebral small vessel disease (cSVD), using amplitude of low-frequency fluctuation (ALFF) in different frequency bands. They found that cSVD patients exhibited significantly lower ALFF, particularly in the cerebellum, hippocampus, and occipital cortex compared to healthy controls, suggesting these regions' involvement in cSVD-related cognitive decline. This research adds to the understanding of cSVD's impact on brain function and its association with cognitive impairment.

The study by Zhao et al. reviews advancements in diagnosing Alzheimer's disease (AD) and mild cognitive impairment (MCI) using 11C-PIB-PET/CT imaging and common neuropsychological tests. It highlights the critical role of early detection and diagnosis through PET/CT imaging in identifying amyloid deposits, which are significant in the pathology of AD and MCI. This approach, combined with neuropsychological assessments, can improve diagnostic accuracy, offering a pathway for early intervention and potentially slowing disease progression.

The study by Zhou et al. provides a comprehensive analysis of hub genes related to oxidative stress in ischemic stroke. Through integrating datasets and employing machine learning methods, they identify key genes and pathways associated with oxidative stress, suggesting potential therapeutic targets. This research underscores the critical role of oxidative stress in stroke pathophysiology and highlights the promise of antioxidant therapy in treatment strategies.

The study by Shen et al. uses functional near-infrared spectroscopy (fNIRS) to examine the effects of focal muscle vibration (FMV) therapy on cortical activity in hemiplegic stroke patients. Specifically, it investigates how FMV applied to the forearm flexor muscles influences cortical regions and correlates with clinical characteristics. The results indicate FMV can activate additional brain cortices, including the prefrontal and sensorimotor areas, potentially supporting its use in stroke rehabilitation to enhance motor function and neural plasticity.

## Author contributions

BH: Writing – original draft, Writing – review & editing.

